# Double trouble: visceral leishmaniasis in twins after traveling to Tuscany – a case report

**DOI:** 10.1186/s12879-018-3394-0

**Published:** 2018-10-01

**Authors:** Charlotte Adamczick, Alexa Dierig, Tatjana Welzel, Alexandra Schifferli, Johannes Blum, Nicole Ritz

**Affiliations:** 1 German Association for Tropical Paediatrics and International Child Health, Frankfurt, Germany; 2 University Children’s Hospital Basel, Paediatric Infectious Diseases and Vaccinology, University of Basel, Basel, Switzerland; 3 University Children’s Hospital Basel, Department of Haematology/Oncology, University of Basel, Basel, Switzerland; 4 Swiss Public Health and Tropical Institute, University of Basel, Basel, Switzerland; 5 University Children’s Hospital Basel, Paediatric Pharmacology, University of Basel, Basel, Switzerland; 6 Department of Paediatrics, The University of Melbourne, Parkville, Australia; 7 Infectious Diseases and Vaccinology, University Children’s Hospital Basel, Spitalstrasse 33, CH-4031 Basel, Switzerland

**Keywords:** Liposomal amphotericin B, Sand fly, Asymptomatic carrier

## Abstract

**Background:**

Leishmaniasis is endemic in many countries worldwide, with a prevalence of 12 million people infected, and an estimated annual incidence of 500 000 visceral leishmaniasis cases. In Europe visceral leishmaniasis is considered endemic mainly in the Mediterranean countries and cases in non-endemic European countries north of the Alps have primarily been reported in returning travellers. The incubation period is typically described between 6 weeks to 6 months. The cases presented highlight the occurrence of longer incubation periods and illustrate the individual variability for progression from infection to disease.

**Case presentation:**

We report the cases of 18-months-old twin girls living at the German-Swiss border, who developed visceral leishmaniasis 7 and 15 months after travelling to Tuscany. They presented with fever of unknown origin and pancytopenia. Both had splenomegaly and in the first case haemophagocytic lymphohistiocytosis or leukaemia was initially included in the differential diagnosis. Diagnosis of visceral leishmaniasis was confirmed by presence of intracytoplasmic localised leishmania parasites on bone marrow aspirate and/or positive leishmania serology. Both girls responded well to treatment with liposomal amphotericin B. The mother and two older siblings remained uninfected, while the father was diagnosed to be an asymptomatic carrier.

**Conclusion:**

Visceral leishmaniasis is an important differential diagnosis for fever of unknown origin and pancytopenia in young children living in countries with endemic disease and highlights the importance of obtaining a detailed travel history. Hemophagocytic lymphohistiocytosis and acute leukaemia present with similar symptoms and consequently are important differential diagnoses. Factors determining progression from infection to disease are not fully understood but younger age seems to be an important risk factor. Screening of siblings from affected individuals therefore may be warranted.

## Background

Leishmaniasis is endemic in at least 88 countries worldwide, with a prevalence of 12 million people infected, and an estimated annual incidence of 500 000 visceral leishmaniasis cases. The World Health Organization has estimated that the annual incidence of visceral leishmaniasis in European countries was approximately 410 to 620 cases between 2003 and 2008 [1]. In Europe visceral leishmaniasis is considered endemic mainly in the Mediterranean countries and the majority of cases are reported to be from Albania, Italy and Spain.

*Leishmania infantum* and *Leishmania donovani* are the main species causing visceral leishmaniasis with *L. infantum* being the most prevalent subspecies in Europe. Humans, rodents and canids are reservoirs and infection follows a bite by an infected female sand fly (*Phlebotomus* spp.). After inoculation of the 15–25 μm long parasite into the tissue or blood stream it is taken up by macrophages which accumulate into lymphatic tissue including spleen, liver and bone marrow. The course of disease depends on the immune status of the host and the specific *Leishmania* spp. [2].

In the following, we are describing the cases of two young children with visceral leishmaniasis after travelling to Tuscany in Northern Italy. The cases presented highlight the occurrence of longer than typically described incubation periods and illustrate the individual variability for progression from infection to disease in genetically related individuals. We also discuss key differential diagnoses and the specific diagnostic and therapeutic approaches used in both cases.

## Case presentation

### First case

An 18-months-old twin girl was referred to our hospital for evaluation of fever of unknown origin in January 2014. She had previously been seen by her paediatrician for daily fever up to 40 °C for one week. Empirical treatment for presumed bacterial infection with a first-generation cephalosporin did not lead to defervescence. No other symptoms including cough, vomiting, diarrhoea, skin rash or weight loss were reported. There were no sick contacts or exposure to pets. The girls’ previous medical history was unremarkable. She had travelled to Tuscany for a three-week holiday six months earlier. The girl’s twin sister, two older siblings, aged four and six years and the parents were well at the time of her presentation.

**Fig. 1:**
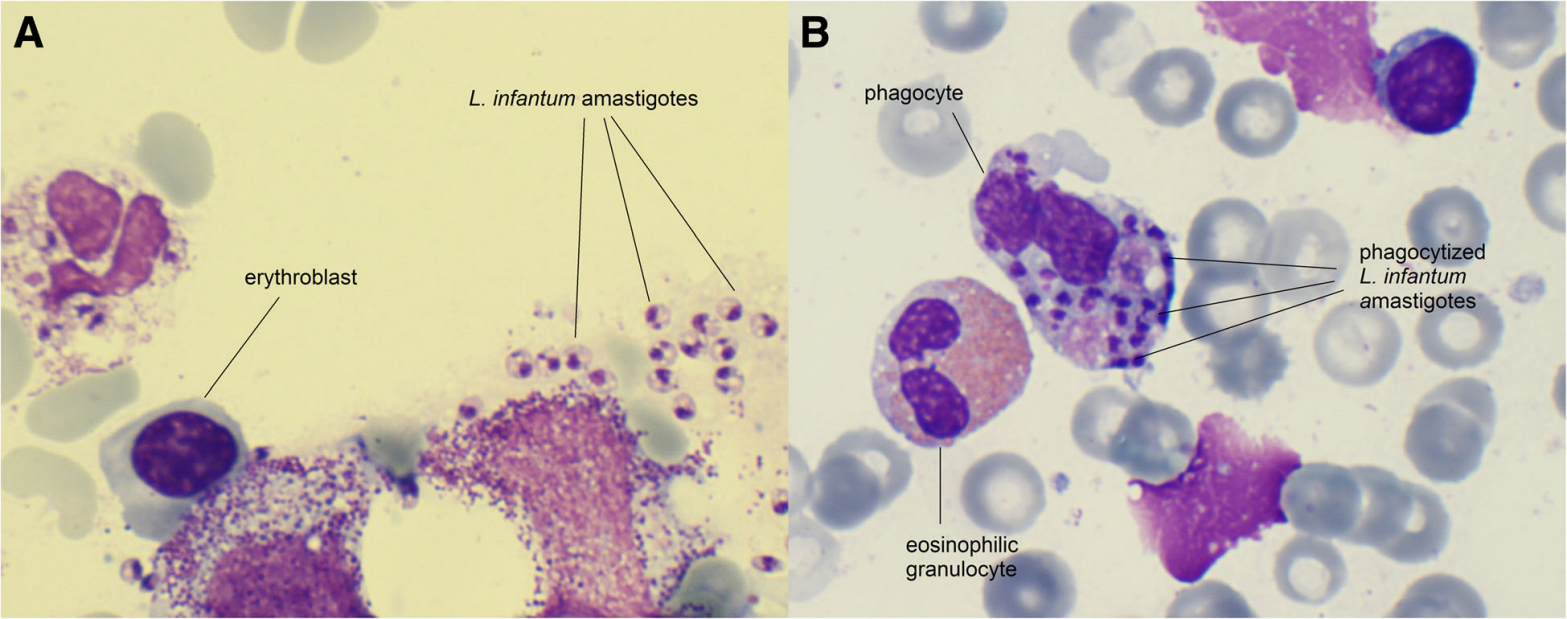
Haematoxylin and eosin stained bone marrow aspirate of the 18-months-old twin (case 1) showing extra- (**a**) and intracellular (**b**) *Leishmania* amastigotes

On physical examination, the girl’s weight and height was 9.9 kg (10th percentile) and 84 cm (75th percentile). She was pale and febrile (38.9 °C), without a focus of infection on clinical examination. Splenomegaly noted on clinical examination was confirmed by ultrasound with a spleen size of 9.9 cm (normal size for age < 9 cm). Her chest radiography was normal. Laboratory investigations (normal values in brackets) showed: haemoglobin 75 (105–135) g/l, platelets 50 (150–450) × 10^9^/l, white blood cells 2.6 (6–17.5) × 10^9^/l with 1.75% of suspected atypical cells, C- reactive protein (CRP) 73 (< 5) mg/l and erythrocyte sedimentation rate (ESR) of 47 (3–13) mm/h. Liver function tests were abnormal for aspartate aminotransferase (ASAT) 145 (26–55) U/L, alanine aminotransferase (ALAT) 80 (9–15) U/L, lactatdehydrogenase (LDH) 1096 (< 338) U/l, and normal for gamma-glutamyltransferase (GGT) and alkaline phosphatase (AP). Renal function tests were normal. Serology was negative for cytomegalovirus, parvovirus B19, Epstein-Bar virus, human herpes virus 8, human immunodeficiency virus (HIV), hepatitis A and toxoplasmosis. Further investigations for haemophagocytic lymphohistiocytosis (HLH) showed a ferritin of 9667 (9–107) μg/l, triglycerides of 1.57 (0,7-0,8) mmol/l and interleukin 2-receptor levels of 7950 (< 800) IU/ml. For suspicion of leukaemia or HLH, a bone marrow aspiration was performed which showed a normal distribution of lymphocyte subsets with predominant T-cells but revealed intracytoplasmic localized *Leishmania* parasites (Fig. 1).

Serology for leishmaniasis was strongly positive with an antibody titre of 1:1280 (cut-off < 1:80) and was performed with an in-house immunofluorescence antibody test (IFA) using liver sections of *Leishmania donovani* infected hamster. In brief, diluted serum was applied to the sections, counter stained with a fluorescein isothiocyanate-labeled conjugate and examined with a fluorescence microscope. Antibodies elicited by all members of the *L. donovani* complex are cross-reactive to the *L. donovani* IFA. Identification and differentiation of *Leishmania* spp. was performed from a bone marrow aspirate by polymerase chain reaction amplification of the Miniexon with subsequent sequencing and revealed infection with *Leishmania infantum* [3, 4].

Treatment with liposomal amphotericin B was initiated with 5 mg/kg on the first day, followed by 3 mg/kg on each day 2–4 and 10. She received a red blood cell transfusion on day three. The girl tolerated the treatment well and her clinical condition gradually improved. She defeveresced after the fourth treatment dose and was discharged on day 7 with the fifth dose (day 10) administered at the district hospital. At follow-up 1.5 years after diagnosis the girl was well with *Leishmania* IFA titre of 1:320.

### Second case

Nine month later (in September 2014), the twin-sister developed fever up to 40 °C with rhinitis. There were no sick contacts. As her sister, she had travelled to Tuscany for a three-weeks holiday 16 months earlier but not returned to Tuscany afterwards or travelled to any other country considered endemic for visceral leishmaniasis. Laboratory investigations at the district hospital showed a haemoglobin of 79 g/l, platelets of 60 × 10^9^/l, white blood cells of 4.9 × 10^9^/l and an elevated LDH of 398 IU/l. Because of pancytopenia and the twin sisters’ previous diagnosis, the girl was referred to our hospital.

On physical examination the girl’s weight was 12.2 kg (25th percentile) and height was 89 cm (50th percentile). She was afebrile and presented with splenomegaly of 11.2 cm in an abdominal ultrasound. Further laboratory investigations showed a CRP of 67 mg/l, normal liver and renal parameters, and negative serology for HIV. *Leishmania* IFA was positive with a titre of 1: 1280 (in-house *L. donovani* IFA as described above) and polymerase chain reaction amplification of the Miniexon sequence from blood confirmed infection with *L. infantum.*

Treatment with liposomal amphotericin B (5 mg/kg/d) was started on the first day followed by 3 mg/kg/d each on day 2–5, 10 and 21. She received a red blood cell transfusion on day 3 because of further haemoglobin decline to 67 g/l. Thrombocytes and white blood cells increased on day four of admission and the girl was discharged on day six with subsequent doses given as an outpatient. At the follow-up 1 year after diagnosis the girl was thriving well and the *Leishmania* IFA had decreased to 1:320.

### Evaluation of the family

Testing of the remaining asymptomatic family members revealed a *Leishmania* IFA of 1:640 for the father (in-house *L. donovani* IFA as described above). He remained asymptomatic, did not receive treatment but is regularly followed the at the outpatient clinic of the Swiss Tropical and Public Health Institute in Basel. The mother and the two older siblings remained asymptomatic with negative *Leishmania* IFA test results.

## Discussion and conclusion

Occurrence of visceral leishmaniasis in Tuscany has been known since many decades but recent reports suggest an increased incidence in the year 2000 in Italy compared to earlier years [5]. Factors assumed to be responsible for this increase remain incompletely understood but challenges in the control of the vector and animal reservoir may have contributed [5]. The specific sand fly species capable to transmit *Leishmania* spp. are now also widely distributed in central and northern Europe [6] potentially leading to autochthonous cases [7]. In addition to travel, the current increased migration of refugees and asylum seekers to Europe may lead to an increase in visceral leishmaniasis incidence as many individuals either originate from regions with high risk or have to cross states known to be visceral leishmaniasis endemic areas [8, 9].

Visceral leishmaniasis is rarely seen in children in central and northern Europe and cases may primarily be investigated for other presumed diseases. Acute leukaemia and hemophagocytic lymphohistiocytosis (HLH) are the two most important differential diagnoses, since symptoms are similar including pancytopenia, hepatosplenomegaly, lymphadenopathy, fever and elevated liver enzymes. Findings suggestive of HLH are hypertriglyceridaemia, elevated ferritin and soluble interleukin 2-receptor levels, but diagnosis may be challenging [10, 11]. Visceral leishmaniasis may trigger HLH and should therefore be excluded before starting immunosuppressive therapy [10, 11].

The incubation period is usually described between 6 weeks to 6 months but may extend from 10 days up to 10 years [12]. Factors that determine progression from infection to visceral leishmaniasis and the interval between infection and development of disease are not fully understood. Interestingly, epidemiological studies from several countries suggest that the majority of individuals with *Leishmania* infection remain asymptomatic [13]. The host’s ability to raise a cell-mediated immune response, notably the balance between Th1 and Th2 immune response seems important [14]. While a Th1 immune response is associated with control of infection, disease progression is observed together with a Th2 immune response [15]. The importance of the cellular immune response is further highlighted by the fact that HIV coinfection is contributing to the rise in severe courses of visceral leishmaniasis [12]. Innate immunity including genetic host factors such as macrophage activation may also play a role for progression of disease [2]. Age seems to be an important additional risk factor for progression as children below 5 years of age are worldwide the most affected age group [12, 16]. This may explains why in the family described the two youngest members progressed to visceral leishmaniasis whereas the father remained asymptomatic. It is debateable, however - if family members under the age of 5 years should be screened for visceral leishmaniasis. Interestingly, both children had rather long incubation periods of 7 and 15 months respectively. This is noteworthy, as despite the girls’ being genetically closely related and infected at the same point in time, the interval of occurrence of clinical symptoms varied substantially.

Treatment recommendations for visceral leishmaniasis are depending on the causative *Leishmania* spp., geographic region, local development of resistance, co-morbidities and immunity of the host and economical resources of the respective health care system [12]. An Albanian study in children reported meglumine antimoniate to be the first line treatment for children with visceral leishmaniasis between 1995 and 2009 with cure rates close to 100% [8]. Contrary to this, other countries in the Mediterranean region and the United States Food and Drug Administration (FDA) recommend liposomal amphotericin B as first line treatment [17]. Recommendations for doses, intervals and duration of treatment vary however considerably. While the FDA recommends a cumulative dose of 21 mg/kg liposomal amphotericin B over 7 administrations (day 1–5, 14, 21) [17] the WHO recommends a cumulative dose 18–21 mg/kg over a 3–6 days period [12]. Further to this, a study in Greece showed that even shorter regimens with a cumulative dose of 20 mg/kg liposomal amphotericin B given over 2 days had high cure rates of 98% [18]. The variability of available recommendations, the absence of a European consensus on the treatment of visceral leishmaniasis in children and the fact that different teams cared for the patients likely explain the difference in treatment regimens reported in the two cases here.

In conclusion, visceral leishmaniasis is an important differential diagnosis in children with fever of unknown origin, pancytopenia and splenomegaly. Careful history taking inducing travel history is key for a high index of suspicion. Specific investigations including serology for leishmaniasis and bone marrow aspirates are important to confirm or rule-out the diagnosis. Longer than classically described incubation periods may occur and a screening of family members with the same exposure particularly for children under 5 years of age should be considered.

## Abbreviations

ALAT: Alanine aminotransferase
AP: Alkaline phosphatase
ASAT: Aspartate aminotransferase
CRP: C-reactive protein
ESR: Erythrocyte sedimentation rate
FDA: United States Food and Drug Administration
GGT: Gamma-glutamyltransferase
HIV: Human immunodeficiency virus
HLH: Haemophagocytic lymphohistiocytosis
IFA: Immunofluorescence antibody test
LDH: Lactatdehydrogenase

## References

[1] Alvar J, Velez ID, Bern C, Herrero M, Desjeux P, Cano J, Jannin J, den Boer M, Team WHOLC (2012). Leishmaniasis worldwide and global estimates of its incidence. PLoS One.

[2] Blackwell JM (1996). Genetic susceptibility to leishmanial infections: studies in mice and man. Parasitology.

[3] Van der Auwera G, Ravel C, Verweij JJ, Bart A, Schonian G, Felger I (2014). Evaluation of four single-locus markers for Leishmania species discrimination by sequencing. J Clin Microbiol.

[4] Marfurt J, Nasereddin A, Niederwieser I, Jaffe CL, Beck HP, Felger I (2003). Identification and differentiation of Leishmania species in clinical samples by PCR amplification of the miniexon sequence and subsequent restriction fragment length polymorphism analysis. J Clin Microbiol.

[5] Gramiccia M, Scalone A, Di Muccio T, Orsini S, Fiorentino E, Gradoni L (2013). The burden of visceral leishmaniasis in Italy from 1982 to 2012: a retrospective analysis of the multi-annual epidemic that occurred from 1989 to 2009. Euro Surveill.

[6] Aspock H, Gerersdorfer T, Formayer H, Walochnik J (2008). Sandflies and sandfly-borne infections of humans in Central Europe in the light of climate change. Wien Klin Wochenschr.

[7] Naucke TJ, Menn B, Massberg D, Lorentz S (2008). Sandflies and leishmaniasis in Germany. Parasitol Res.

[8] Petrela Raida, Kuneshka Loreta, Foto Eli, Zavalani Ferit, Gradoni Luigi (2010). Pediatric Visceral Leishmaniasis in Albania: A Retrospective Analysis of 1,210 Consecutive Hospitalized Patients (1995–2009). PLoS Neglected Tropical Diseases.

[9] Pohl C, Mack I, Schmitz T, Ritz N (2017). The spectrum of care for pediatric refugees and asylum seekers at a tertiary health care facility in Switzerland in 2015. Eur J Pediatr.

[10] Bode SF, Bogdan C, Beutel K, Behnisch W, Greiner J, Henning S, Jorch N, Jankofsky M, Jakob M, Schmid I (2014). Hemophagocytic lymphohistiocytosis in imported pediatric visceral leishmaniasis in a nonendemic area. J Pediatr.

[11] Henter JI, Horne A, Arico M, Egeler RM, Filipovich AH, Imashuku S, Ladisch S, McClain K, Webb D, Winiarski J (2007). HLH-2004: diagnostic and therapeutic guidelines for hemophagocytic lymphohistiocytosis. Pediatr Blood Cancer.

[12] WHO expert commitee on the control of Leishmaniases: Control af the Leishmaniases. In*.*; 2010.

[13] Michel G, Pomares C, Ferrua B, Marty P (2011). Importance of worldwide asymptomatic carriers of Leishmania infantum (L. chagasi) in human. Acta Trop.

[14] Roberts MT (2005). Current understandings on the immunology of leishmaniasis and recent developments in prevention and treatment. Br Med Bull.

[15] Bogdan C, Rollinghoff M (1998). The immune response to Leishmania: mechanisms of parasite control and evasion. Int J Parasitol.

[16] Alawieh A, Musharrafieh U, Jaber A, Berry A, Ghosn N, Bizri AR (2014). Revisiting leishmaniasis in the time of war: the Syrian conflict and the Lebanese outbreak. Int J Infect Dis.

[17] Aronson N, Herwaldt BL, Libman M, Pearson R, Lopez-Velez R, Weina P, Carvalho EM, Ephros M, Jeronimo S, Magill A (2016). Diagnosis and treatment of Leishmaniasis: clinical practice guidelines by the Infectious Diseases Society of America (IDSA) and the American Society of Tropical Medicine and Hygiene (ASTMH). Clin Infect Dis.

[18] Syriopoulou V, Daikos GL, Theodoridou M, Pavlopoulou I, Manolaki AG, Sereti E, Karamboula A, Papathanasiou D, Krikos X, Saroglou G (2003). Two doses of a lipid formulation of amphotericin B for the treatment of Mediterranean visceral leishmaniasis. Clin Infect Dis.

